# The impact of clinical and laboratory parameters on clinical pregnancy and live birth rates in fresh cycles: a retrospective study of 9608 high-quality cleavage-stage embryos

**DOI:** 10.1186/s13048-024-01371-x

**Published:** 2024-02-21

**Authors:** Haijing Zhao, Keer Gan, Xiaohui Ji, Lingyan Zheng, Songbang Ou, Mei Mei, Qingxue Zhang, Hui Chen, Ping Yuan, Wenjun Wang

**Affiliations:** 1 IVF Center, Department of Obstetrics and Gynecology, Sun Yat-sen Memorial Hospital, Sun Yat-sen University, 107 Yanjiang Xi Road, Guangzhou, Guangdong 510120 China; 2 Guangdong Provincial Clinical Research Center for Obstetrical and Gynecological Diseases, Guangzhou, China; 3 Medical College, Jinan University, Guangzhou, Guangdong 510632 China

**Keywords:** High-quality embryo, Blastocyst, Clinical pregnancy rate, Live birth rate

## Abstract

**Background:**

Evidence from the Istanbul consensus workshop suggests correlations between morphological parameters and embryo developments. 8-cell embryos are the best blastomere stage on day 3. No good quality evidence exists to support high-quality embryonic selection following blastulation and clinical outcomes. This study aimed to investigate the factors that affect blastocyst formation, blastocyst quality, and clinical outcomes of high-quality cleavage-stage embryos in fresh cycles.

**Methods:**

This study was a retrospective analysis of 9608 high-quality cleavage-stage embryos from 2987 couples between January 2017 to June 2021, namely 1520 embryos categorized as “812” (8-cell, grade 2, mild fragmentation), 2961 as “821” (8-cell, grade 2, mild asymmetry), 896 as “711” (7-cell, grade 1), and 517 as “911” (9-cell, grade 1) compared with 3714 embryos categorized as “811” (8-cell, grade 1). The primary outcomes were clinical pregnancy rate (CPR) and live birth rate (LBR). Blastulation rate (BR), available late blastocyst rate (ABR) and high-quality late blastocyst rate (HBR) were secondary outcome measures.

**Results:**

BR, ABR, and HBR had significant differences among the five groups (*P* < 0.001), while CPR and LBR were also significantly different in cleavage-stage fresh transfer (*P* < 0.01). The multivariable multilevel logistic regression analysis revealed a significant association between cell number, cell size, blastocyst development and clinical outcomes. For 7 to 9-cell highest-quality embryo, mild fragmentation and more blastomeres were more conducive to blastocyst formation and clinical outcomes. While cleavage-stage embryos developed into blastocysts, the negative impact of their initial morphology on clinical outcomes would be erased.

**Conclusions:**

Our study firstly evaluated blastocyst development and clinical outcomes of high-quality cleavage-stage embryos in fresh cycles, with rankings of 811, 812, 911, 821, and 711. We found the initial morphological characteristics of the high-quality cleavage-stage embryos did not adversely impact clinical outcomes, even as they progressed to the blastocyst stage.

**Supplementary Information:**

The online version contains supplementary material available at 10.1186/s13048-024-01371-x.

## Introduction

Human pre-implantation embryonic development comprises a series of dynamic morphological changes and the appearance of particular morphological features at specific time points, which can be observed by light microscopy. Fertilization, cleavage, and blastocyst stages are three vital points of observation during in vitro fertilization (IVF). In two-pronuclear (2PN) zygote derived normal developing cleavage embryos range from the 2-cell stage to the morula, the blastomere undergoes cell division every 18–20 h [1]. Ideally, if all blastomeres underwent division in exact synchrony, all embryos observed from day 1 to day 4 after fertilization would comprise 2, 4, 8, or 16 cells. However, asynchronous development is a common phenomenon, resulting in different cell numbers, different blastomere sizes, and different degrees of fragmentation [1].

Generally, embryos with less than 10% fragmentation, stage-specific cell size, and no multinucleation, especially 7- to 9-cell embryos on day 3, are categorized as grade 1 cleavage embryos [2]. The 8-cell grade 1 embryo on day 3 is considered a highest-quality cleavage-stage embryo. Increasing fragmentation and non-stage specific cell sizes are known to result in reduced blastulation and cell differentiation, while increasing cell numbers are positively correlated with implantation and live birth rates [1, 3]. Therefore, cleavage-stage embryo scoring systems are mainly based on the evaluation of cell number, cell size, and the degree of fragmentation, and do not generally include multinucleation. However, the most influential one among these affecting embryo development factors has not been clearly determined so far.

Blastocyst quality during blastulation is an important indicator to reflect the developmental potential of cleavage-stage embryos. Therefore, blastocyst culture has been used as a method to select the most viable embryos [4]. High-quality embryos generally exhibit appropriate kinetics and develop into high-quality blastocysts. However, there have been some reports of high-quality cleavage embryos failing to undergo blastulation, which has been considered to occur due to influence from other factors such as female age and the use of different assisted reproductive technologies (ART) [5, 6]. Most of these previous studies were based on univariate analyses, and therefore lacked insights that could be provided by overall multi-factor analyses. A study involving multiple logistic regression analysis that evaluated various components of day 3 embryo morphology found that the live birth rate was negatively associated with maternal age, increasing fragmentation, and asymmetry scores [7]. Despite fragmentation or asymmetry being present only to a mild extent, choosing the appropriate cleavage site embryo transfer or blastocyst culture for IVF was still a perplexing issue. Hence, as our research objective, we sought to determine among the aforementioned factors, those that played the key role, and those that played a secondary role in blastulation and clinical outcomes of high-quality embryos on day 3, which could be used to evaluate the success rate of transfer or blastocyst culture and to provide the single embryo transfer strategy in high-quality cleavage-stage embryos cohort.

In this study, high-quality embryos on day 3 were divided into five groups for the analysis of blastulation rate, available late blastocyst, high-quality blastocyst, clinical pregnancy and live birth. We evaluated the effect of different factors, including the effect of cell number differing by one, mild fragmentation, mild asymmetry, male and female age, female body mass index (BMI), female basic follicle stimulating hormone (FSH) and luteinizing hormone (LH) levels, the duration and type of infertility, ovulation stimulation protocol, ART method, total oocyte pick-up numbers, endometrium thickness and progesterone on trigger day by comparing the other four groups with an 8-cell grade 1 group. We also compared the fresh blastocysts transfer outcomes from high-quality embryos on day 3 between the five groups.

## Materials and methods

### Study subjects

This retrospective cohort study, conducted from January 2017 to June 2021, involved 9608 embryos from 2987 couples evaluated for blastulation and clinical outcomes. The inclusion criteria were defined as: the 2PN-derived high-quality embryos on day 3 with cell numbers between 7 and 9, symmetrical stage-specific cell size, less than 10% fragmentation and no multinucleation; and the 2PN-derived high-quality embryos on day 3 with 8 cells with either 10–25% fragmentation or stage-specific cell size for the majority of cells, and no multinucleation. Supplementary Table 1 showed the characteristics of patients belong to different high-quality cleavage embryos groups. The time of embryo culture lasted until the fifth or the sixth day after ovum pick up according to the state of blastocyst formation. The couples having cycles of rescue intracytoplasmic sperm injection (ICSI), egg or sperm donation, oocyte cryopreservation, and artificial oocyte activation were excluded. For fresh embryo transfer cycle, endometrium thickness less than 7 mm or more than 20 mm, progesterone more than 1.5 ng/mL on trigger day were excluded.

### Embryo culture and evaluation

**Fig. 1:**
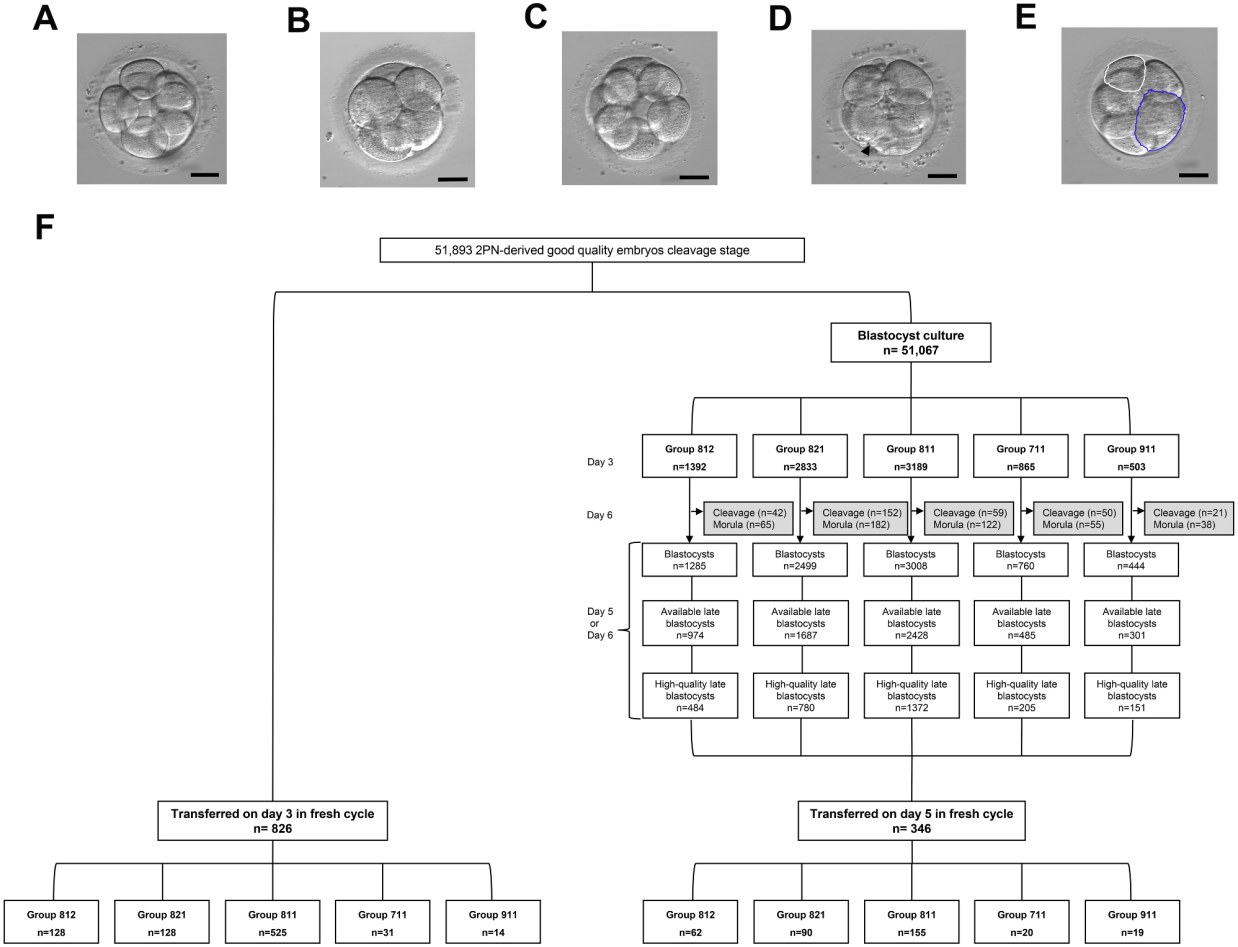
High-quality cleavage-stage embryo characteristics and flow diagram of retrospective study selection for blastocyst culture and embryo transfer. Phenotypes of high-quality cleavage-stage embryos examined by light microscopy. Scale bars 50 μm. (**A**) category ‘811’ was defined to include embryos with 8 cells and symmetrical stage-specific cell size and less than 10% fragmentation. (**B**) category ‘711’ was defined to include embryos with 7 cells and symmetrical stage-specific cell size and less than 10% fragmentation. (**C**) category ‘911’ was defined to include embryos with 9 cells and symmetrical stage-specific cell size and less than 10% fragmentation. (**D**) category ‘812’ was defined to include embryos with 8 cells and symmetrical stage-specific cell size and 10 − 25% fragmentation. The black arrowhead indicates fragmentation. (**E**) category ‘821’ was defined to include embryos with 8 cells and asymmetrical stage-specific cell size (the white circle and the blue circle) for minority of cells (1–2 cells) and less than 10% fragmentation. (**F**) Flow diagram of this study selection for blastocyst culture and embryo transfer of five groups

The oocytes were assessed for fertilization at approximately 20 h post-injection based on the presence of two pronuclear and the second polar body. Embryos were cultured in G1-plus/G2-plus sequential media (Vitrolife, Sweden) in a humidified atmosphere containing 5% O_2_ and 6% CO_2_. Following the observation time point and criteria established by the Istanbul consensus workshop on embryo assessment [2], cleavage-stage embryos on day 3 were assessed at 68 ± 1 h after fertilization. Embryos having no multinucleation were assessed according to the indices of cell numbers, stage-specific cell size, and fragmentation. Accordingly, the scoring format was replaced by a numerical shorthand having a sequential representation of cell number, stage-specific cell size, and fragmentation (e.g., 8-cell, grade 1 embryos were categorized as ‘811’; 8-cell, grade 2 embryos with stage-specific cell size for the majority of cells were categorized as ‘821’; 8-cell, grade 2 embryos with fragmentation were categorized as ‘812’) [2]. To evaluate the effect of the factors related to the cleavage-stage embryo on blastulation and clinical outcomes in terms of different morphological parameters, the cleavage-stage embryo score groups were subdivided into five categories: category ‘811’ was defined to include embryos with 8 cells and symmetrical stage-specific cell size and less than 10% fragmentation, which were considered as the standard for the best high-quality embryos (Fig. 1A); category ‘711’ was defined to include embryos with 7 cells and symmetrical stage-specific cell size and less than 10% fragmentation, having one cell less than ‘811’ embryos (Fig. 1B); category ‘911’ was defined to include embryos with 9 cells and symmetrical stage-specific cell size and less than 10% fragmentation, having one cell more than ‘811’ embryos (Fig. 1C); category ‘812’ was defined to include embryos with 8 cells and symmetrical stage-specific cell size and 10–25% fragmentation, which was more than that of ‘811’ embryos (Fig. 1D); category ‘821’ was defined to include embryos with 8 cells and asymmetrical stage-specific cell size for minority of cells (1–2 cells), and less than 10% fragmentation, which could be considered a mildly asymmetrical stage-specific cell size than that of ‘811’ embryos (Fig. 1E). The first single fresh embryo transfer was performed for the high-quality cleavage-stage embryos and them-derived blastocysts (Fig. 1F). Based on the Gardner grading system [8], blastocysts were classified as early and late blastocysts. Blastocysts graded as 1–2 were categorized as early blastocysts, and blastocysts graded as 3–6 were categorized as late blastocysts [8]. Among the late blastocysts, those with either the inner cell mass (ICM) or trophectoderm (TE) graded above C were categorized as available late blastocysts (AB). In the late blastocyst groups, those with ICM and TE scores ≥ BB were categorized as high-quality late blastocyst (HB) including 3/4/5/6AA, AB, BA, or BB. A particular embryo that was still in cleavage or morula stage on day 6 was considered to undergo embryonic developmental arrest. That was all enrolled high-quality cleavage embryos on day 3 being in blastocyst culture to day 6 except it had formed available late blastocysts on day 5 (Fig. 1F). All embryo scorings were checked by two experienced embryologists.

### Study design and outcome parameters

We collected 2PN-derived high-quality embryos on day 3 to evaluate factors affecting blastocyst formation and quality and to provide the single embryo transfer strategy in high-quality cleavage-stage embryos cohort. Firstly, based on criteria set by the Istanbul consensus workshop on embryo assessment [2], in this study, 8-cell, grade 1 embryo which was the highest-quality embryo on day 3 was categorized as ‘811’ as the gold standard for morphological comparison. Therefore, the high-quality embryo cohort were divided into five groups according to cell number, cell size and fragmentation, comprising 3189 embryos in group 811, 2833 embryos in group 821, 1392 embryos in group 812, 865 embryos in group 711, and 503 embryos in group 911 (Fig. 1F), in order to evaluate the effect of embryonic morphological parameters by comparing the rate of embryos arrest in cleavage stage and the rate of blastulation (B) and available late blastocyst (AB) and the high-quality blastocyst (HB). Secondly, we analyzed the effect of embryonic morphological parameters combined with other factors including embryonic parental age, female BMI which calculated as weight in kilograms divided by height in meters squared, female serum basic FSH and LH levels, the duration and type of infertility, ovulation stimulation protocol, ART method, and total oocyte pick-up numbers using univariate and multivariable multilevel logistic regression. When *P*-value is less than 0.1 in univariate multilevel logistic regression model, variables would be further analyzed in multivariate multilevel stepwise regression model. Ovulation stimulation protocols were divided into four protocols including gonadotropin-releasing hormone (GnRH) agonist, GnRH antagonist, mild stimulation and natural cycle. ART methods were divided into four methods according to sperm quality parameters and genetic factors including conventional IVF, ICSI, percutaneous epididymal sperm aspiration (PESA) or testicular sperm aspiration (TESA), and preimplantation genetic testing (PGT). In our center, conventional IVF procedure was used for the sufficient number of progressively motile sperm for insemination (the progressively motile sperm concentration ranging between 0.1 and 0.5 × 10^6^/ml) after density gradient centrifugation [9]. ICSI method was used for severe male factors (including severe oligospermia, severe asthenospermia, severe teratozoospermia, and abnormal sperm acrosin activity) or fertilization rate less than 30% in previous conventional IVF cycle. PESA or TESA was used for azoospermatism. The semen analysis was performed according to the protocols described in the World Health Organization (WHO) manual [10]. PGT, based on ICSI technology in our center, was used for genetic factors including chromosomal and monogenic abnormality. The type of infertility comprised primary and secondary infertility. Thirdly, we investigated the clinical outcomes of transferred embryos on day 3 and day 5 in different five groups.

The blastulation rate (BR) is defined as the number of blastocysts divided by the number of cleavage embryos cultured on day 3. The available late blastocyst rate (ABR) is defined as the number of available late blastocysts divided by the number of cleavage embryos cultured on day 3. The high-quality late blastocyst rate (HBR) is defined as the number of high-quality late blastocysts divided by the number of all blastocysts. The rate of arrest in cleavage stages is defined as the number of embryos arrest in cleavage stage divided by the number of cleavage embryos cultured on day 3. The clinical pregnancy rate (CPR) is defined as the number of patients with ultrasonographic visualization of one or more gestational sacs four to seven weeks after transplantation or definitive clinical signs of pregnancy divided by the transferred cycles. The live birth rate (LBR) is defined as the number of infants divided by the transferred cycles.

### Statistical analysis

All statistical procedures were conducted using SPSS version 23.0. Data are presented as mean ± standard deviation (SD) for continuous variables and are summarized by frequency and percentage for categorical variables. Comparisons between groups were performed using the Chi-square test, Fisher’s exact test, and Kruskal-Wallis test for categorical variables. One-way analysis of variance was used to assess the difference between groups for continuous variables. Pairs of groups were compared using Bonferroni test for categorical variables. Considering the correlations of two or more embryos derived from a couple, multilevel mixed-effects logistic regression model at the patient levels were applied to calculated Odds ratios (ORs) and 95% confidence intervals (CIs) in univariate and multivariate analysis (Fig. 3). Forest plots of the effect of risk factors on blastocyst formation and quality, and stack histograms of main outcomes were plotted using R version 4.2.2. All statistical tests were two-tailed, and a *P*-value < 0.05 was considered statistically significant.

**Fig. 2:**
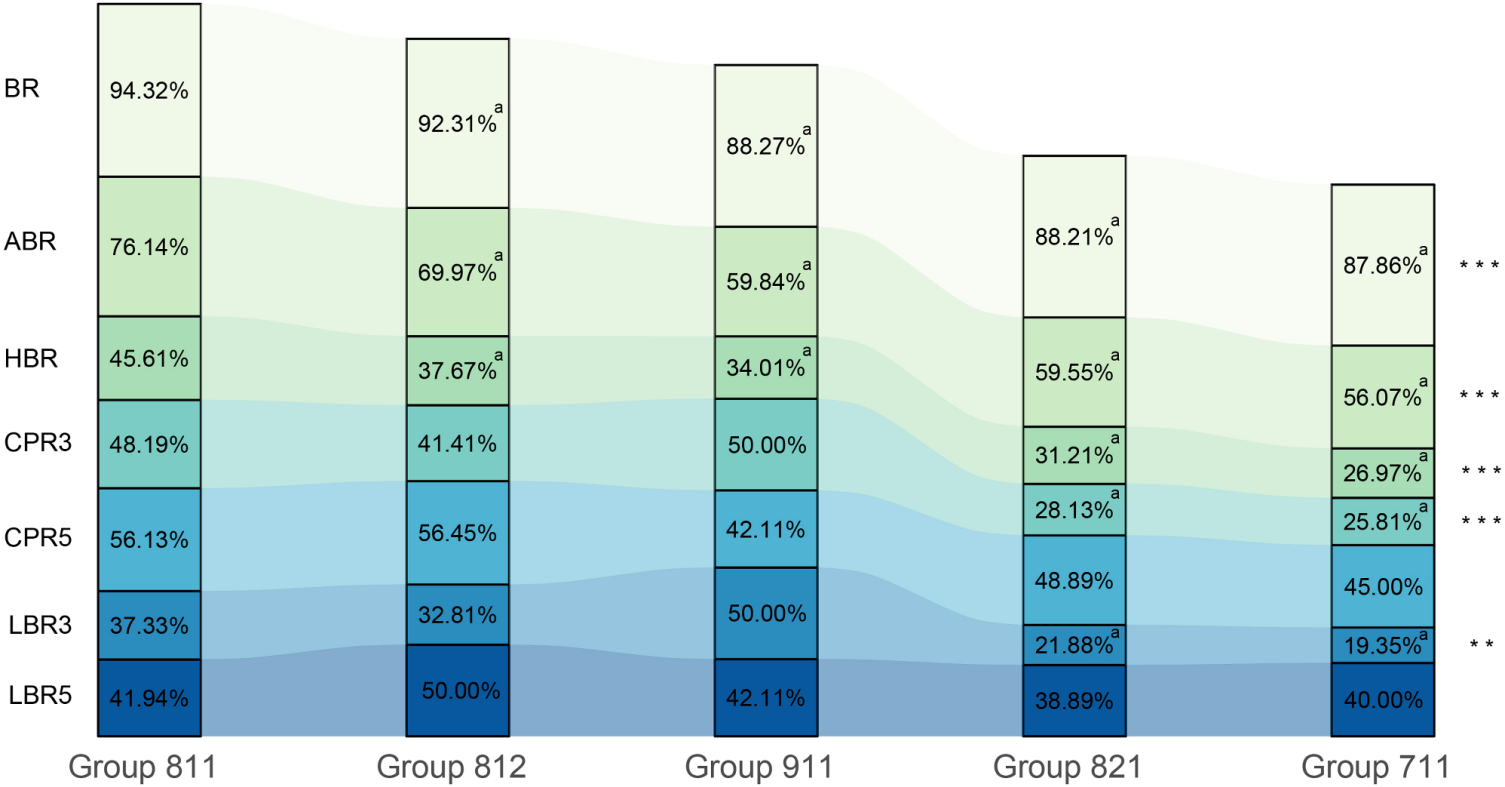
Rates of blastocyst culture and main clinical outcomes in different five groups. Statistical significance was defined as a *P*-value < 0.05. *** *P* < 0.001, **0.001 ≤ *P* < 0.01,* 0.01 ≤ *P* < 0.05. ^a^ Statistical significance compared with group 811

**Fig. 3:**
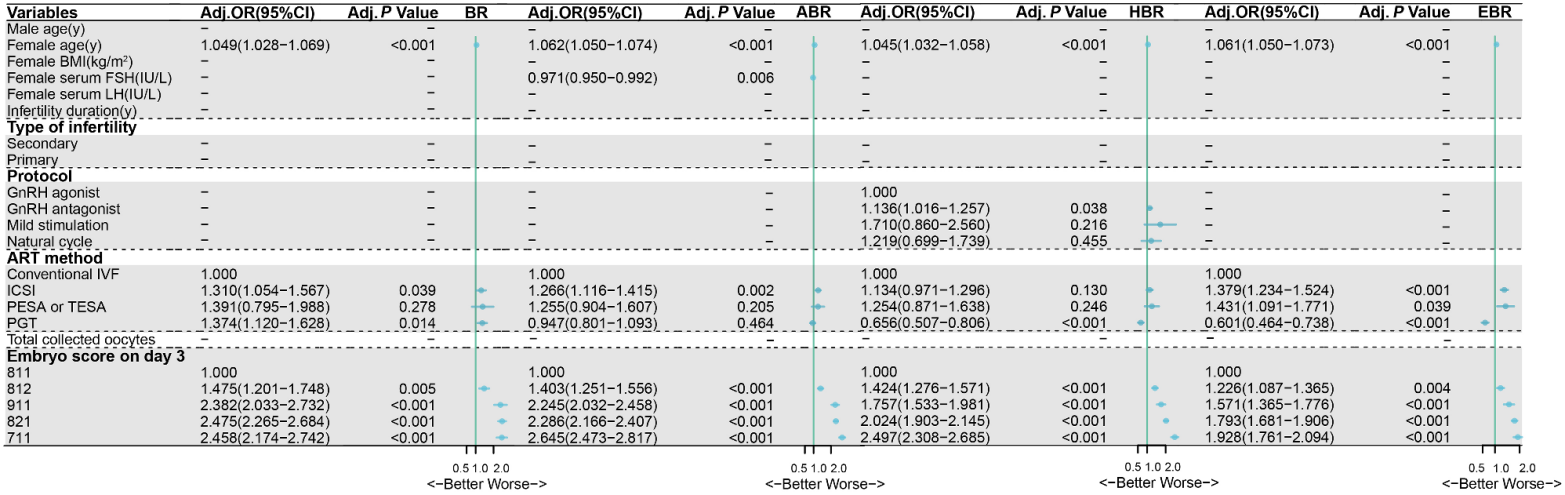
Forest plot of studies of multivariate multilevel logistic regression analyses of the effect on blastocysts, available late blastocysts, high-quality blastocysts and expanded available late blastocysts on day 5. Statistical significance was defined as a *P*-value < 0.05. *Abbreviations* ABR, available late blastocyst rate; Adj., adjust; ART, assisted reproductive technology; BR, blastulation rate; BMI, body mass index; CI, confidence interval; EBR, expended available late blastocyst rate on day 5; FSH, follicle stimulating hormone; GnRH, gonadotropin-releasing hormone; HBR, high-quality blastocyst rate; LH, luteinizing hormone; IVF, in vitro fertilization; ICSI, intracytoplasmic sperm injection; OR, odds ratio; PESA, percutaneous epididymal sperm aspiration; PGT, preimplantation genetic testing; TESA, testicular sperm aspiration

## Results

The study included 9602 high-quality cleavage-stage embryos, in which 8782 embryos were subjected to blastocyst culture and 826 cleavage embryos were performed for single fresh embryo transfer on day 3 (Fig. 1F).

### Blastocyst culture

The BR, ABR, and HBR were 91.05% (7996/8782) and 66.90% (5875/8782) and 37.42% (2992/7996), respectively. Group 711 had the highest rate of arrest in cleavage stages, followed by groups 821, 911, 812, and 811 (*P* < 0.001). In contrast, group 811 had the highest BR, ABR, and HBR, followed by groups 812, 911, 821, and 711 (*P* < 0.001) (Fig. 2). This implied that embryonic development and blastulation were associated with cell number, cell size, and fragmentation, and mild fragmentation (less than 10%) had less effect on embryonic development than mild asymmetry when evaluated at the same blastomeres. If the effects of asymmetry and fragmentation are excluded, high-quality embryo ‘911’ can be observed to be beneficial for blastulation than ‘711’.

The rate and expansion stage of AB on day 5 were analyzed to evaluate the rate of blastocyst development in different groups. The ABR on day 5 was higher for group 911 than that of the other two groups (75.42% vs. 74.46% vs. 55.88%), with a statistically significant difference (*P* < 0.001), but no significant differences were observed between the ABRs of any of the 8-cell groups (Table 1). This implies that mild asymmetry and fragmentation might not have a significant effect on the ABR on day 5 when evaluated at the same blastomeres, whereas increasing the cell number might accelerate blastocyst development. Furthermore, the same parameters for AB graded as 4 or above (≥ 4) in terms of blastocyst expansion were evaluated. Group 911 was found to have the highest ABR correlated with expansion stage, followed by groups 811 and 711 (*P* = 0.001) (Table 1). However, when evaluated at the same blastomeres, asymmetry had a more negative effect on expanded AB compared with fragmentation (*P* = 0.018) (Table 1).

**Table 1:** Analysis of the available late blastocysts from different cleavage stages on different days and expansion stages

Variable		Day 5, n (%)		Day 6, n (%)	*P* ^a^
**Same cell numbers**					
group 811			1808 (74.46)	620 (25.54)	0.891
	expansion stage ≥ 4	1286 (71.13)			0.018
	expansion stage < 4	522 (28.87)			
group 821			1245 (73.80)	442 (26.20)	
	expansion stage ≥ 4	826 (66.35)			
	expansion stage < 4	419 (33.65)			
group 812			723 (74.23)	251 (25.77)	
	expansion stage ≥ 4	505 (69.85)			
	expansion stage < 4	218 (30.15)			
**Different cell numbers**					
group 811			1808 (74.46)	620 (25.54)	< 0.001
	expansion stage ≥ 4	1286 (71.13)			0.001
	expansion stage < 4	522 (28.87)			
group 711			271 (55.88)	214 (44.12)	
	expansion stage ≥ 4	165 (60.89)			
	expansion stage < 4	106 (39.11)			
group 911			227 (75.42)	74 (24.58)	
	expansion stage ≥ 4	167 (73.57)			
	expansion stage < 4	60 (26.43)			

^a^ Kruskal-Wallis test

The characteristics of patients and comparison in the five different groups are presented in Supplementary Table 1. Based on univariate and multivariate multilevel logistic regression analyses, the embryo score on day 3 was found to be the most significant factor related to blastulation, followed by female age and the ART method (Fig. 3). The significant factors for AB were female age, female serum basic FSH level, ICSI, and embryo score on day 3 (Fig. 3). The significant factors for HB were female age, protocol, ART method, and embryo score on day 3 (Fig. 3). The significant factors for expanded AB on day 5, were female age, ART method, and embryo score on day 3 (Fig. 3). Collectively, asymmetry and lower cell number in the cleavage stage and female advanced age were main to negatively affect the formation and quality of blastocysts.

### Clinical outcomes after SET

There were 826 cycles for a single cleavage embryo transfer on day 3 and 346 cycles for a single blastocyst transfer on day 5 (Fig. 1F). Among day 3 transfer cycles, group 911 had the highest CPR and LBR among all the transferred embryos, followed by groups 811, 812, 821, and 711 (*P* < 0.001 and *P* = 0.004, respectively) (Fig. 2) (Supplementary Table 2). The rates of biochemical pregnancy, miscarriage, ectopic pregnancy, infant sex, birth weight, and body length had no significant differences among the five groups. Surprisingly, CPR and LBR in fresh blastocysts transfer on day 5 had no significant differences among these five groups (Fig. 2) (Supplementary Table 2).

**Fig. 4:**
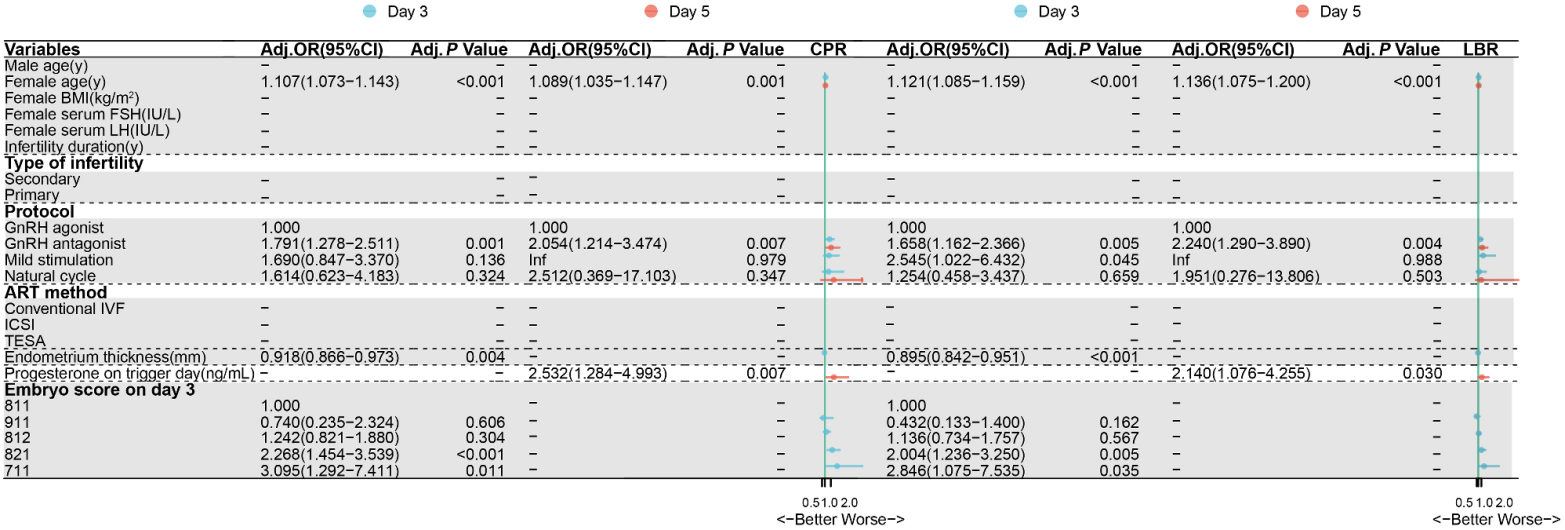
Forest plot of studies of multivariate multilevel logistic regression analyses of the effect on the clinical pregnancy and live birth outcomes on day 3 and day 5. Statistical significance was defined as a *P*-value < 0.05. *Abbreviations* Adj., adjust; ART, assisted reproductive technology; BMI, body mass index; CI, confidence interval; CPR, clinical pregnancy rate; FSH, follicle stimulating hormone; GnRH, gonadotropin-releasing hormone; LBR, live birth rate; LH, luteinizing hormone; IVF, in vitro fertilization; ICSI, intracytoplasmic sperm injection; OR, odds ratio; PESA, percutaneous epididymal sperm aspiration; TESA, testicular sperm aspiration

The characteristics of patients with all transferred embryos were shown in Supplementary Table 3. For a single cleavage embryo transfer on day 3, the embryo score on day 3 (asymmetry and lower cell number), female age, protocol (GnRH antagonist), and progesterone on trigger day were effective factors for clinical pregnancy and live birth (Fig. 4). However, for a single blastocyst transfer on day 5, female age, protocol (GnRH antagonist), and progesterone on trigger day were effective factors for clinical pregnancy and live birth, and the effect of embryo score on day 3 would be absent (Fig. 4).

## Discussion

In this study, we evaluated the effect of embryonic morphology and maternal age on blastulation and clinical outcomes including pregnancy and live birth outcomes in high-quality cleavage-stage embryos, while provide the single high-quality embryo transfer strategy when choosing a day 3 or day 5 embryo transfer. Our results also revealed that blastocyst culture was crucial to clinical outcomes even if for the high-quality cleavage-stage embryos. The clinical outcomes had significant differences especially in asymmetric 8-cell and symmetric 7-cell embryos before blastocyst culture, however, the five groups were no significant differences after blastocyst culture.

Parameters related to embryonic morphology are crucial indicators for ensuring good quality embryo choice. With regard to human pre-implantation embryos, prevailing views suggest that 7–9 blastomeres on day 3 represent the optimal number of cells for normal embryos, of which 8-cell embryos are considered as the most representative group in scoring system of day 3. Thus, 8-cell embryos are given the highest preference for transplantation in comparison with 7-cell or 9-cell embryos on day 3. We suggest that for ensuring proper selection of good quality embryos, besides cell number, other factors affecting embryo development should be considered, and an order of preference can be determined based on analyzing these factors even for 7–9-cell embryos when we needed do embryo transfer on day 3. Our study identified cell number, cell size, and fragmentation to be major factors of blastocyst development, and advanced age in females as a secondary factor. We found less than 10% fragmentation have less effect on blastocyst development and clinical outcome than mild asymmetry when evaluating at the same blastomeres. Moreover, embryos in group 911 should be given higher priority to be selected for IVF compared to group 711, since the higher cell number makes the embryo more conducive to blastulation in case of the other factors being equal.

Based on the Istanbul consensus workshop on embryo assessment, our scoring system for cleavage-stage embryos included scoring of cell number, cell size, fragmentation, and multinucleation [2]. A previous study revealed that the live birth rate of embryos transferred on day 3 was positively associated with increasing cell number for embryos that reached up to the 8-cell stage and was negatively associated with greater fragmentation percentage and increasing asymmetry [7]. For cell numbers other than 8 (including more than or less than 8), the live birth rate has been reported to decrease [7]. There also exists a consensus that embryos undergoing cleavage slower or faster than normal have a reduced implantation potential [2]. Thus, 8-cell embryos are generally considered to be of better quality compared to that of embryos having cell numbers other than 8 on day 3. It is thus necessary for other morphological parameters to be considered for being incorporated into the scoring system. The order of importance of the three parameters (cell number, cell size, and fragmentation) in our study was also shown to be important for scoring of the embryos. Our study compared the blastocyst formation rate in four groups having values of parameters differing slightly among them with the group 811. Based on the results the three main inferences with regard to embryo quality were as follows: (1) The 8-cell grade 2 (mild fragmentation) embryos were superior to 9-cell grade 1 embryos; (2) The 9-cell grade 1 embryos were superior to 8-cell grade 2 (mild asymmetry) embryos; and (3) The 8-cell grade 2 (mild asymmetry) embryos were superior to 7-cell grade 1 embryos (Fig. 2). Moreover, our multiple multilevel logistic regression analyses also identified the order of importance of the above-mentioned factors, which consistently influenced BR, ABR, and HBR (Fig. 3). Thus, it could be inferred that mild fragmentation had less effect on blastulation than that of cell number (addition or subtraction of single blastomere), while the cell number (addition or subtraction of single blastomere) had less effect than that of cell size (mild asymmetry) in high-quality cleavage-stage embryos.

At the AB stage, the cell numbers were greater than or equal to 85, while in expanded AB (≥ 4), cell numbers approached 130 on day 5 [11]. Day 5 blastocysts had a greater preference for IVF transfer than day 6 blastocysts for both, fresh as well as frozen cycles, due to the former being associated with significantly higher clinical pregnancy and live birth rates [12]. In addition, the expanded AB are known to be positively associated with clinical implantation rates [13, 14]. Therefore, cleavage embryos with satisfactory developmental potential and speed were considered to be associated with the rate of expanded AB on day 5. In our study, 8-cell embryos on day 3 showed no significant differences in the formation rate of day 5 AB, but showed significant differences in the formation rate of day 5 expanded AB. The loss of a blastomere can be seen to affect the expanded AB formation (Table 1; Fig. 2). Our multiple multilevel logistic regression analysis showed embryo score on day 3, ART method, and female age to be factors with a significant influence on day 5 expanded AB, with embryo score being the main factor (Fig. 3).

After fresh cleavage-stage embryos transfer, the rates of pregnancy and live birth showed the similar outcomes to the blastocysts development of high-quality day 3 embryos (Fig. 2). The blastocyst formation was associated with the ranking of day 3 embryo score across the 5 groups, while the quality of blastocysts had an impact on birth indicators. It implied that the blastocyst development could predict the trend toward clinical outcomes. The differences of clinical outcomes disappeared when embryos had developed into blastocysts, while the quality and grade of transfer blastocysts had no significant differences among five groups (Supplementary Table 4). During human pre-implantation embryos, the gene translation mainly came from maternal transcripts before 8-cell embryos, while zygotic genome activation got started after 8 cells [15, 16]. It implied that the inner factors of embryos themselves played a minor role as long as the formation of blastocysts and the activation of zygotic genome in comparison of other maternal factors. A study also provided evidences of strong self-repair ability of cleavage embryos, showing that they can develop into euploid blastocysts even after experiencing abnormal cleavage [17]. Therefore, we assumed that all embryos reached the same starting point at the blastocyst stage, as the cleavage score is no longer relevant to complex cellular organization and developmental potential. Apart from embryonic factors, maternal factors such as female age, endocrine factors, uterine microenvironment, and progesterone levels on trigger day continue to have an impact on pregnancy outcomes.

Progesterone more than 1.5 ng/mL on trigger day was associated with lower LBR in multiple clinical studies [18, 19]. High concentration of progesterone would induce premature transition of the endometrium and affect endometrial receptivity. However, progesterone less than 1.5 ng/mL still impacted the clinical outcomes in fresh blastocyst transfer in our study (Fig. 4). A previous study revealed that CPR with progesterone less than 1.0 ng/mL was significantly higher than progesterone with 1.0 to 1.5 ng/mL [20], while another study showed significantly lower CPR with less than 0.5 ng/mL [18]. Meanwhile, progesterone was a dynamic parameter during the process of assisted reproduction, which increasing from the day of oocyte pick-up to the day 3 of embryo culture [21]. However, the levels of progesterone decreased in approximately one-third of patient 3 days after oocyte pick-up [21], which might be related to a decrease in pregnancy rate. Compared with day 3 transfers, more variable factors about progesterone would affect the clinical outcomes of day 5 transfer in fresh cycles.

Advanced age of females is known to affect the number of retrieved oocytes as well as reduce blastulation [22]. Studies have reported advanced age of males to significantly affect pregnancy outcomes and blastocyst formation rates [23, 24]. However, the above studies have not considered the impact of embryo scoring on day 3. The analysis of our dataset showed no significant correlations between advanced age of males and blastulation, in terms of BR, ABR, and HBR. Our results show a negative association between advanced age of females and blastulation, in terms of BR, ABR, and HBR, and expanded AB on day 5, which elucidates the relationship of these parameters for high-quality cleavage-stage embryos. A similar association was also founded between advanced age of females and clinical outcomes. Advanced age in females is known to be associated with decreased ovarian reserve, which leads to increased FSH levels and decreased oocyte collection [25]. Therefore, advanced age of females should be considered as a vital factor associated with blastocyst formation, embryo implantation, and live birth, regardless of cleavage embryos being scored as high-quality.

The usage of ART methods has been speculated to be a key indicator of the efficiency of blastocyst development. A previous study involving a comparative analysis of ART methods showed the ICSI group to have more available blastocysts than the IVF group [6]. However, another study revealed the IVF group to have a higher blastulation rate than that of the ICSI group [26]. The notion of the IVF group having more available late blastocysts than the ICSI group is supported by our data (Fig. 3) which implied that IVF-derived embryos should be prior to transfer than ICSI-derived embryos under the same conditions. Additionally, some studies have suggested that human embryos undergoing blastocyst development arrest are more likely to have abnormal chromosomes [27, 28]. However, a study indicates there to be no detectable effect of chromosomal aberration on morphology at any preimplantation stage in the case of the most clinically relevant aneuploidies [29]. Our results revealed three different correlations involving blastocyst development and ART methods: (1) no significant difference was observed between the preimplantation genetic testing (PGT) and IVF groups with regard to AB formation; (2) the PGT group showed positive correlation with the formation of HB and expanded AB on day 5 compared to that observed in the IVF group; (3) the PGT group showed negative correlation with the blastulation rate compared to that observed in the IVF group (Fig. 3). The inconclusive nature of these observations leads us to highlight the need for further well-designed randomized controlled studies to investigate the relationship of the ART method with blastocyst formation.

In addition, the ovarian stimulation protocols were seen to be no significant effect on BR, ABR and expanded ABR on day 5, whereas HBR, CPR and LBR in GnRH antagonist protocol were lower than that in GnRH agonist protocol. Other studies also found that the number of available embryos in group GnRH agonist was higher than these of group GnRH antagonist in suboptimal responders [30, 31]. A systematic review and meta-analysis including 34 studies of general population showed that group GnRH antagonist had lower CPR than group GnRH agonist, while LBR had no significant differences between two groups [32]. The purpose of GnRH antagonist protocol was to decrease the ovarian hyperstimulation syndrome risk in polycystic ovary syndrome patients. Since clinical outcomes of fresh embryo transfer with antagonist protocol are lower, it is recommended to consider the strategy of frozen embryo transfer, even for the transfer of high-quality cleavage-stage embryos.

The limitation of our study was the lack of dynamic observation of embryos by time-lapse monitoring. However, major IVF centers had been difficult in observing all embryos by time-lapse imaging under above 3,000 IVF cycles every year. Based on big data of 9608 high-quality cleavage-stage embryos, the consistent outcomes of blastocyst development, including BR, ABR and HBR, had clearly clarified the results. In addition, our study provided a primary conclusion about the transfer order of embryos considering clinical pregnancy rate and live birth rate, but further study should be conducted due to the small sample in some groups in transferred embryos.

## Conclusions

In high-quality cleavage-stage embryos, the outcomes of blastulation are highly similar to the clinical outcomes after fresh transfer cycles, ranking by the evaluation of blastocyst development, that is 811, 812, 911, 821, 711, which were associated with the significant effect of asymmetry and lower cell number. While the cleavage-stage embryos developed into blastocysts, the negative impact of their cleavage-stage embryo morphology on clinical outcomes would be erased. Moreover, the endometrium thickness significantly affects the clinical outcome of fresh cleavage stage transfer, while progesterone levels significantly affect the clinical outcome of fresh blastocyst stage embryo transfer. Advanced age of females and GnRH antagonist protocol are always two important factors that needs to be considered for fresh transfer cycle, even when transferring high-quality embryos.

### Electronic supplementary material

Below is the link to the electronic supplementary material.

**Supplementary Material 1**: Supplementary table 1. Characteristics of patients in different cleavage stages in blastocyst culture

**Supplementary Material 2**: Supplementary table 2. Analysis of the clinical outcomes of transferred embryos in different cleavage stages

**Supplementary Material 3**: Supplementary table 3. Characteristics of patients in different cleavage stages in transferred embryos

**Supplementary Material 4**: Supplementary table 4. Analysis of the transferred blastocysts from different cleavage stages in blastocysts grade and quality on day 5

## Data Availability

The data supporting the conclusions of this article are included within the article and its supplementary files.

## Abbreviations

BR: blastulation rate
ABR: available late blastocyst rate
HBR: high-quality late blastocyst rate
CPR3: clinical pregnancy rate of cleavage embryos
CPR5: clinical pregnancy rate of blastocysts
LBR3: live birth rate of cleavage embryos
LBR5: live birth rate of blastocysts
