# Supplementary material for: The impact of clinical and laboratory parameters on clinical pregnancy and live birth rates in fresh cycles: a retrospective study of 9608 high-quality cleavage-stage embryos
Source: J Ovarian Res. 2024 Feb 21;17:47. doi: 10.1186/s13048-024-01371-x (PMC10882753; doi:10.1186/s13048-024-01371-x)
Supplement: Supplementary file 1 — Supplementary Material 1: Supplementary table 1. Characteristics of patients in different cleavage stages in blastocyst culture [file 13048_2024_1371_MOESM1_ESM.docx]

| **Supplementary table 1. Characteristics of patients in different cleavage stages in blastocyst culture** | | | | | | |
| --- | --- | --- | --- | --- | --- | --- |
| **Characteristic** | **Group 811 (n=3189)** | **Group 821 (n=2833)** | **Group 812 (n=1392)** | **Group 711 (n=865)** | **Group 911 (n=503)** | ***P*^a^** |
| Male age (y) | 33.86±5.23 | 33.62±5.25 | 33.84±5.26 | 33.73±5.42 | 33.68±5.09 | >0.05 |
| Female age (y) | 31.59±4.54 | 31.42±4.46 | 31.80±4.46 | 31.64±4.45 | 31.53±4.04 | >0.05 |
| Female BMI (kg/m^2^) | 21.55±2.89 | 21.46±2.82 | 21.59±3.28 | 21.47±2.94 | 21.52±3.19 | >0.05 |
| Female serum FSH (IU/L) | 7.47±2.52 | 7.43±2.85 | 7.48±2.49 | 7.47±2.53 | 7.62±3.05 | >0.05 |
| Female serum LH (IU/L) | 5.77±4.07 | 5.93±4.36 | 5.94±4.23 | 5.95±4.73 | 6.13±4.44 | >0.05 |
| Female serum E_2_ (ng/L) | 61.60±2.12 | 59.42±2.28 | 64.05±3.22 | 60.33±4.23 | 60.00±4.53 | >0.05 |
| AMH (ng/mL) | 6.12±4.30 | 6.00±3.51 | 5.98±4.24 | 5.97±4.31 | 6.25±4.30 | >0.05 |
| Infertility duration (y) | 3.57±2.90 | 3.57±2.71 | 3.72±2.90 | 3.87±2.80 | 3.80±2.81 | >0.05 |
| Type of infertility |  |  |  |  |  |  |
| Primary | 1962 (61.52) | 1108 (39.11) | 570 (40.95) | 410 (47.40) | 202 (40.16) | <0.05^b,c,d,e^ |
| Secondary | 1227 (38.48) | 1725 (60.89) | 822 (59.05) | 455 (52.60) | 301 (59.84) | <0.05^b,c,d,e^ |
| Infertility cause |  |  |  |  |  |  |
| Female factors | 1572(49.29) | 1601(56.51) | 782(56.17) | 478(55.26) | 286(56.86) | <0.05^b,c,d,e^ |
| Male factors | 272(8.53) | 280(9.88) | 117(8.41) | 99(11.45) | 49(9.74) | >0.05 |
| Mutual factors | 516(16.18) | 502(17.72) | 244(17.53) | 170(19.65) | 96(19.09) | >0.05 |
| Genetic factors | 829 (26.00) | 450 (15.89) | 249 (17.89) | 118 (13.64) | 72 (14.31) | <0.05^b,c,d,e^ |
| Protocol |  |  |  |  |  |  |
| GnRH agonist | 2143 (67.20) | 1822 (64.31) | 910 (65.37) | 595 (68.79) | 338 (67.19) | >0.05 |
| GnRH antagonist | 979 (30.70) | 979 (34.56) | 459 (32.98) | 252 (29.13) | 160 (31.81) | <0.05^b,c,e^ |
| Mild stimulation | 19 (0.59) | 10 (0.35) | 6 (0.43) | 8 (0.92) | 3 (0.60) | >0.05 |
| Natural cycle | 48 (1.51) | 22 (0.78) | 17 (1.22) | 10 (1.16) | 2 (0.40) | >0.05 |
| ART method |  |  |  |  |  |  |
| Conventional IVF | 1918 (60.14) | 1901 (67.10) | 934 (67.10) | 554 (64.05) | 342 (67.99) | <0.05^b,c,d,e^ |
| ICSI | 385 (12.07) | 410 (14.47) | 182 (13.07) | 169 (19.54) | 76 (15.11) | <0.05^d,e^ |
| PESA or TESA | 57 (1.79) | 72 (2.54) | 27 (1.94) | 24 (2.77) | 13 (2.58) | >0.05 |
| PGT | 829 (26.00) | 450 (15.89) | 249 (17.89) | 118 (13.64) | 72 (14.31) | <0.05^b,c,d,e^ |
| Total collected oocytes | 18.23±8.58 | 18.89±7.92 | 18.11±7.71 | 18.17±8.04 | 17.95±7.68 | <0.05^b^ |
| Abbreviations: AMH, anti-Mullerian hormone; ART, assisted reproductive technology; BMI, body mass index; E_2_, estradiol; FSH, follicle stimulating hormone; GnRH, gonadotropin-releasing hormone; LH, luteinizing hormone; IVF, in vitro fertilization; ICSI, intracytoplasmic sperm injection; PESA, percutaneous epididymal sperm aspiration; PGT, preimplantation genetic testing; TESA, testicular sperm aspiration.  Data are presented as the number (%) or mean ± SD.  a Kruskal-Wallis test. b 821 vs. 811. c 812 vs. 811.  d 711 vs. 811.  e 911 vs. 811. Statistical significance was defined as a *P* value < 0.05. | | | | | | |
