# Supplementary material for: The impact of clinical and laboratory parameters on clinical pregnancy and live birth rates in fresh cycles: a retrospective study of 9608 high-quality cleavage-stage embryos
Source: J Ovarian Res. 2024 Feb 21;17:47. doi: 10.1186/s13048-024-01371-x (PMC10882753; doi:10.1186/s13048-024-01371-x)
Supplement: Supplementary file 2 — Supplementary Material 2: Supplementary table 2. Analysis of the clinical outcomes of transferred embryos in different cleavage stages [file 13048_2024_1371_MOESM2_ESM.docx]

**Supplementary table 2. Analysis of the clinical outcomes of transferred embryos in different cleavage stages**

| **Variable** | **Day 3** | | | | | | **Day 5** | | | | | | |
| --- | --- | --- | --- | --- | --- | --- | --- | --- | --- | --- | --- | --- | --- |
|  | **Group 811**  **(n=525)** | **Group 821**  **(n=128)** | **Group 812**  **(n=128)** | **Group 711**  **(n=31)** | **Group 911**  **(n=14)** | ***P*^a^** | | **Group 811**  **(n=155)** | **Group 821**  **(n=90)** | **Group 812**  **(n=62)** | **Group 711**  **(n=20)** | **Group 911**  **(n=19)** | ***P*^a^** |
| Clinical pregnancies rate  (per ET cycle)(n,%) | 253(48.19) | 36(28.13) | 53(41.41) | 8(25.81) | 7(50.00) | ＜0.001^b^ | | 87(56.13) | 44(48.89) | 35(56.45) | 9(45.00) | 8(42.11) | 0.571 |
| Biochemical pregnancies rate  (per ET cycle)(n,%) | 24(4.57) | 6(4.69) | 2(1.56) | 1(3.23) | 0(0) | 0.507 | | 11(7.10) | 2(2.22) | 4(6.45) | 2(10.00) | 1(5.26) | 0.496 |
| Miscarriage rate  (per clinical pregnancy)(n,%) | 53(20.95) | 7(19.44) | 10(18.87) | 2(25.00) | 0(0) | 0.760 | | 19(21.84) | 7(15.91) | 4(11.43) | 1(11.11) | 0(0) | 0.389 |
| Ectopic pregnancies rate  (per clinical pregnancy)(n,%) | 2(0.79) | 1(2.78) | 0(0) | 0(0) | 0(0) | 0.421 | | 2(2.30) | 2(4.55) | 0(0) | 0(0) | 0(0) | 0.624 |
| Live birth rate  (per ET cycle)(%) | 196(37.33) | 28(21.88) | 42(32.81) | 6(19.35) | 7(50.00) | 0.004 ^b^ | | 65(41.94) | 35(38.89) | 31(50.00) | 8(40.00) | 8(42.11) | 0.740 |
| Male rate  (per live birth)(n,%) | 100(51.02) | 15(53.57) | 26(61.90) | 5(83.33) | 4(57.14) | 0.442 | | 41(63.08) | 20(57.14) | 18(58.06) | 6(75.00) | 6(75.00) | 0.784 |
| Female rate  (per live birth)(n,%) | 96(48.98) | 13(46.43) | 16(38.10) | 1(16.67) | 3(42.86) | 0.442 | | 24(36.92) | 15(42.86) | 13(41.94) | 2(25.00) | 2(25.00) | 0.784 |
| Birth weight  (kg)(mean±SD) | 3.12±0.49 | 3.14±0.42 | 3.08±0.49 | 2.69±0.62 | 3.29±0.36 | 0.312 | | 3.15±0.51 | 3.16±0.40 | 3.11±0.35 | 3.08±0.72 | 3.26±0.61 | 0.887 |
| Body length  (cm)(mean±SD) | 49.70±1.88 | 49.61±2.59 | 49.56±3.85 | 48.60±1.52 | 49.40±1.95 | 0.348 | | 49.76±3.08 | 49.50±2.64 | 49.04±2.44 | 48.00±3.74 | 49.80±3.03 | 0.796 |

a Kruskal-Wallis test.

b 821 vs. 811.
