# Supplementary material for: The impact of clinical and laboratory parameters on clinical pregnancy and live birth rates in fresh cycles: a retrospective study of 9608 high-quality cleavage-stage embryos
Source: J Ovarian Res. 2024 Feb 21;17:47. doi: 10.1186/s13048-024-01371-x (PMC10882753; doi:10.1186/s13048-024-01371-x)
Supplement: Supplementary file 3 — Supplementary Material 3: Supplementary table 3. Characteristics of patients in different cleavage stages in transferred embryos [file 13048_2024_1371_MOESM3_ESM.docx]

| **Supplementary table 3. Characteristics of patients in different cleavage stages in transferred embryos** | | | | | | |  |
| --- | --- | --- | --- | --- | --- | --- | --- |
| **Characteristic** | **Group 811 (n=680)** | **Group 821 (n=218)** | **Group 812 (n=190)** | **Group 711 (n=51)** | **Group 911 (n=33)** | ***P*** ^a^ |  |
| Male age (y) | 35.57±6.14 | 35.94±6.01 | 35.63±5.90 | 34.75±5.40 | 34.36±6.47 | >0.05 |  |
| Female age (y) | 33.15±5.09 | 33.79±5.26 | 33.65±5.48 | 32.51±4.81 | 32.52±5.82 | >0.05 |  |
| Female BMI (kg/m^2^) | 21.79±3.01 | 21.86±2.84 | 21.90±3.22 | 21.55±2.84 | 22.73±3.72 | >0.05 |  |
| Female serum FSH (IU/L) | 8.30±4.71 | 8.48±3.77 | 8.43±4.09 | 7.47±2.25 | 7.89±2.34 | >0.05 |  |
| Female serum LH (IU/L) | 5.32±4.43 | 4.98±3.28 | 5.69±4.02 | 5.75±6.02 | 5.37±4.23 | >0.05 |  |
| Infertility duration (y) | 4.01±3.29 | 4.24±3.25 | 4.24±3.21 | 4.38±3.58 | 2.97±2.20 | >0.05 |  |
| Type of infertility |  |  |  |  |  |  |  |
| Primary | 234 (34.41) | 68 (31.19) | 78 (41.05) | 23 (45.10) | 11 (33.33) | >0.05 |  |
| Secondary | 446 (65.59) | 150 (68.81) | 112 (58.95) | 28 (54.90) | 22 (66.67) | >0.05 |  |
| Infertility cause |  |  |  |  |  |  |  |
| Female factors | 443(65.15) | 135(61.93) | 131(68.95) | 31(60.78) | 13(39.39) | >0.05 |  |
| Male factors | 86(12.65) | 31(14.22) | 20(10.53) | 7(13.73) | 8(24.24) | >0.05 |  |
| Mutual factors | 151(22.20) | 52(23.85) | 39(20.52) | 13(25.49) | 12(36.36) | >0.05 |  |
| Protocol |  |  |  |  |  |  |  |
| GnRH agonist | 434 (63.82) | 136 (62.39) | 121 (63.69) | 36 (70.59) | 22 (66.67) | >0.05 |  |
| GnRH antagonist | 202 (29.71) | 58 (26.61) | 48 (25.26) | 11 (21.57) | 8 (24.24) | >0.05 |  |
| Mild stimulation | 31 (4.56) | 16 (7.34) | 15 (7.89) | 4 (7.84) | 2 (6.06) | >0.05 |  |
| Natural cycle | 13 (1.91) | 8 (3.66) | 6 (3.16) | 0 (0) | 1 (3.03) | >0.05 |  |
| ART method |  |  |  |  |  |  |  |
| Conventional IVF | 539 (79.26) | 172 (78.90) | 158 (83.16) | 33 (64.71) | 25 (75.76) | >0.05 |  |
| ICSI | 119 (17.50) | 36 (16.51) | 24 (12.63) | 15 (29.41) | 7 (21.21) | >0.05 |  |
| PESA or TESA | 22 (3.24) | 10 (4.59) | 8 (4.21) | 3 (5.88) | 1 (3.03) | >0.05 |  |
| Endometrium thickness(mm) | 11.61±2.55 | 11.38±2.59 | 11.62±2.68 | 11.26±2.47 | 11.24±2.39 | >0.05 |  |
| Progesterone on trigger day(ng/mL) | 0.85±0.33 | 0.86±0.35 | 0.82±0.32 | 0.89±0.31 | 0.74±0.31 | >0.05 |  |
| Abbreviations: ART, assisted reproductive technology; BMI, body mass index; FSH, follicle stimulating hormone; GnRH, gonadotropin-releasing hormone; LH, luteinizing hormone; IVF, in vitro fertilization; ICSI, intracytoplasmic sperm injection; PESA, percutaneous epididymal sperm aspiration; TESA, testicular sperm aspiration.  Data are presented as the number (%) or mean ± SD.  a Kruskal-Wallis test.  Statistical significance was defined as a *P* value < 0.05. | | | | | | | |
