# Supplementary material for: The impact of clinical and laboratory parameters on clinical pregnancy and live birth rates in fresh cycles: a retrospective study of 9608 high-quality cleavage-stage embryos
Source: J Ovarian Res. 2024 Feb 21;17:47. doi: 10.1186/s13048-024-01371-x (PMC10882753; doi:10.1186/s13048-024-01371-x)
Supplement: Supplementary file 4 — Supplementary Material 4: Supplementary table 4. Analysis of the transferred blastocysts from different cleavage stages in blastocysts grade and quality on day 5 [file 13048_2024_1371_MOESM4_ESM.docx]

**Supplementary table 4. Analysis of the transferred blastocysts from different cleavage stages in blastocysts grade and quality on day 5**

| **Variable** | **expansion stage ≥ 4, n (%)** | | | **expansion stage < 4, n (%)** | ***P*^a^** | **high-quality, n (%)** | **non-high-quality, n (%)** | ***P*^a^** |
| --- | --- | --- | --- | --- | --- | --- | --- | --- |
| Group 811 | 116 (74.84) | | | 39 (25.16) | 0.182 | 112 (72.26) | 43 (27.74) | 0.057 |
| Group 812 | 42 (67.74) | | | 20 (32.26) |  | 40 (64.51) | 22 (35.48) |  |
| Group 821 | 56 (62.22) | | | 34 (37.78) |  | 52 (57.78) | 38 (42.22) |  |
| Group 711 | 11 (55.00) | | | 9 (45.00) |  | 9 (45.00) | 11 (55.00) |  |
| Group 911 | 13 (68.42) | | | 6 (31.58) |  | 12 (63.16) | 7 (36.84) |  |
| ^a^ Kruskal-Wallis test. | |  |  |  |  |  |  |  |
